# Immune-related adverse events correlate with the efficacy of PD-1 inhibitors combination therapy in advanced cholangiocarcinoma patients: A retrospective cohort study

**DOI:** 10.3389/fimmu.2023.1141148

**Published:** 2023-03-24

**Authors:** Yanfang Zhang, Xiaoting Wang, Yinyan Li, Yun Hong, Qingwei Zhao, Ziqi Ye

**Affiliations:** ^1^ Department of Clinical Pharmacy, the First Affiliated Hospital, Zhejiang University School of Medicine, Hangzhou, China; ^2^ Department of Medical Oncology, the First Affiliated Hospital, Zhejiang University School of Medicine, Hangzhou, China; ^3^ Zhejiang Provincial Key Laboratory for Drug Evaluation and Clinical Research, the First Affiliated Hospital, Zhejiang University School of Medicine, Hangzhou, China

**Keywords:** advanced cholangiocarcinoma, PD-1 inhibitors combination therapy, immune-related adverse events (irAEs), efficacy prediction, clinical marker

## Abstract

**Background:**

Whether irAEs can predict the efficacy of PD-1 inhibitors in cholangiocarcinoma (CCA) has not been assessed. Therefore, this study aims to investigate the correlation between irAEs and the therapeutic effect of PD-1 inhibitors combination therapy in patients with advanced CCA.

**Methods:**

All patients with CCA who were consecutively admitted to the inpatient unit of our hospital and received PD-1 inhibitors combination therapy between September 2020 and April 2022 were screened. In total, 106 patients with CCA were screened out. We then followed up these patients until October 2022. Due to perioperative use (n=28), less than 2 cycles of PD-1 inhibitor therapy (n=9), incomplete data (n=8) and no pathological report (n=2), 59 patients were included in the final analysis. The patients were divided into the irAEs cohort and the non-irAEs cohort according to whether they experienced irAEs or not. The Log-Rank test was performed to compare the difference in survival time between these two cohorts. We then applied multivariate COX regression analysis to investigate whether irAEs were independent prognostic factors for survival in patients with advanced CCA.

**Results:**

Finally, 32 patients were included in the irAEs cohort and 27 patients in the non-irAEs cohort. A total of 32 patients (54.2%) had any-grade irAEs, of which 4 patients (6.8%) had grade 3-4 irAEs. The most common irAEs were thyroid toxicity (30.5%) and dermatologic toxicity (30.5%). There were no notable differences in demographics and clinical characteristics between the irAEs and non-irAEs cohorts, except for total bilirubin level (P=0.026) and relapse (P=0.016). The disease control rate (DCR) in the irAEs cohort was higher than in the non-irAEs cohort (90.6% vs 70.4%, P=0.047). Median overall survival (OS) and median progression-free survival (PFS) were better in the irAEs cohort than in the non-irAEs cohort (OS: 21.2 vs 10.0 months, P<0.001; PFS: 9.0 vs 4.4 months, P=0.003). Multivariate COX regression analysis showed that irAEs were independent prognostic factors for OS and PFS (OS: HR=0.133, 95% CI: 0.039-0.452, P=0.001; PFS: HR=0.435, 95% CI: 0.202-0.934, P=0.033).

**Conclusion:**

IrAEs correlated with improved DCR, OS, and PFS in advanced CCA patients receiving PD-1 inhibitors combination therapy.

## 1 Introduction

Cholangiocarcinoma (CCA) is major type of biliary tract cancer (BTC) that originates from the bile duct epithelium (1). It is the second most common hepatobiliary cancer with high malignancy and poor prognosis (1). Depending on its anatomical location, CCA is divided into extrahepatic cholangiocarcinoma (ECC) and intrahepatic cholangiocarcinoma (ICC). CCA has few treatment options and is insensitive to both radiotherapy and chemotherapy. The onset of CCA is latent and is often at a later stage of the tumor when it is diagnosed (2). Surgery is currently the most effective treatment for CCA. However, only about 35% of patients with early-stage CCA can be treated with radical surgery. Even with radical surgery, patients still have a postoperative relapse rate of up to 70-75% (3, 4). Gemcitabine in combination with cisplatin has been the standard first-line treatment for patients with unresectable or metastatic CCA since 2010, based on the phase 3 ABC-02 trial (5). However, the overall survival (OS) of patients with advanced CCA treated with chemotherapy remains unsatisfactory.

With the ongoing development of precision medicine, immunotherapeutic drugs, particularly immune checkpoint inhibitors (ICIs), have made breakthroughs in cancer treatment. Inhibitors of the programmed cell death protein 1 (PD-1)/programmed death-ligand 1 (PD-L1) play an important therapeutic role in a wide range of tumors (6–9). In the phase 3 TOPAZ-1 trial in 685 patients with advanced BTC, durvalumab (a PD-L1 inhibitor) in combination with gemcitabine and cisplatin improved OS compared to placebo plus chemotherapy (median OS: 12.8 months vs 11.5 months, P=0.021) (10). Therefore, durvalumab plus gemcitabine and cisplatin is recommended as one of the preferred first-line regimens for advanced CCA. The efficacy of PD-1 inhibitors in CCA has also been extensively studied, but objective response rates (ORR) vary widely between studies, ranging from 17.5% to 40.9% (11–14). Therefore, there is a need to identify biomarkers that predict response to PD-1 inhibitors in CCA. Currently, deficient mismatch repair/microsatellite instability-high (dMMR/MSI-H), tumor mutation burden-high (TMB-H) and PD-L1 expression have been widely studied as biomarkers related to response to immunotherapy in solid tumors (15–17), but the evidence in CCA is insufficient (11, 12, 14, 18, 19). Recently, several biomarkers have been identified in ICC to predict the efficacy of PD-1inhibitors, including pre-treatment serum lipid levels, inflammation-based scores and immune-related RNA signatures (20–22). However, there is still a lack of clinical markers during treatment to identify patients who may benefit from PD-1 inhibitors in CCA. Interestingly, PD-1 inhibitors not only activated effector T cells, but also reduced regulatory T cells, which may play a key role in maintaining immune tolerance (23). As a result, the immune system can also attack normal cells, leading to immune-related adverse events (irAEs) (24). Several studies have shown that irAEs can serve as an effective clinical biomarker for PD-1 inhibitors in several cancers, such as lung, liver, gastric cancers, and melanoma (24–28). However, there is a lack of studies on whether irAEs may play a similar role in CCA.

Therefore, this retrospective cohort study aims to investigate the correlation between irAEs and the efficacy of PD-1 inhibitors combination therapy in CCA patients. It will provide a theoretical basis for whether irAEs can be considered as a clinical marker for predicting the efficacy of PD-1 inhibitors combination therapy, and provide evidence for the precise screening of CCA patients who may benefit from PD-1 inhibitors combination therapy.

## 2 Materials and methods

### 2.1 Participants

All CCA patients consecutively admitted to the inpatient unit of our hospital and received PD-1 inhibitors combination therapy between September 2020 and April 2022 were screened. A total of 106 patients with CCA were screened out. A pathology report was required for each patient. All patients were then followed up until October 2022.

### 2.2 Inclusion and exclusion criteria

Inclusion criteria; (1) pathologically diagnosed with CCA; (2) ≥18 years of age, no gender restrictions; (3) received PD-1 inhibitors in combination with chemotherapy or targeted therapy [including multi-target tyrosine kinase inhibitors (TKIs)]; (4) received at least two cycles of PD-1 inhibitors combination therapy; (5) Eastern Cooperative Oncology Group (ECOG) performance status (PS) score was 0-1. Exclusion criteria: (1) incomplete medical records; (2) received PD-1 inhibitor combinations therapy for perioperative treatment.

### 2.3 Measures

We reviewed electronic medical records and extracted demographic information and laboratory parameters of eligible patients, including gender, age, ECOG PS score, TNM stage, total bilirubin, alpha-fetoprotein (AFP), and carcinoembryonic antigen (CEA) before PD-1 inhibitors combination therapy. A fixed dose of 200mg for pembrolizumab, sintilimab, camrelizumab, and tislelizumab and 3 mg/kg or 240 mg for toripalimab was given intravenously every 3 weeks. The choice of PD-1 inhibitor for treatment does not follow strict guidelines, mainly because of the accessibility and price of the drugs. In addition, to date, no studies have shown which PD-1 inhibitor is more effective than others. According to National Comprehensive Cancer Network (NCCN) guidelines, CCA patients who developed irAEs require a decision-making team consisting of oncology, pharmacy, endocrinology, dermatology and pulmonology specialists. This team determined whether the patient needed to discontinue PD-1 inhibitors and received appropriate observation, levothyroxine supplementation, anti-allergy medication, and topical or systemic hormone therapy. Multi-target TKIs combined with PD-1 inhibitors were lenvatinib (8 mg orally per day) or anlotinib (10 mg orally once daily d1-14, every 3 weeks for one cycle), which are recommended by the China Society of Clinical Oncology (CSCO) guidelines for advanced BTC patients. The specific combination regimens for each patient are shown in **Supplementary Table 1**.

Response to treatment was assessed according to the Response Evaluation Criteria in Solid Tumors version 1.1 (RECIST 1.1). ORR, defined as the proportion of patients who achieved a partial response (PR) or complete response (CR). The disease control rate (DCR) was defined as the proportion of patients who achieved not only PR and CR but also stable disease (SD). Progression-free survival (PFS) was defined as the time from the first cycle of PD-1 inhibitors combination therapy until death or disease progression, whichever came first. In addition, OS was defined as the time from the first cycle of PD-1 inhibitors combination treatment to death from any cause. The irAEs were assessed and graded for severity according to the Common Terminology Criteria for Adverse Events 5.0 (CTCAE 5.0). Patients were then divided into an irAEs cohort and a non-irAEs cohort based on whether they had irAEs or not.

### 2.4 Statistical analysis

Data were calculated as mean ± standard deviation (SD) for continuous variables with normal distribution, otherwise as median (interquartile range). Categorical variables were presented as frequencies and percentages. Student’s t-test or Mann-Whitney U test was used to compare differences between the irAEs cohort and the non-irAEs cohort for continuous variables. Fisher’s exact test or Chi-square test was used for categorical variables. Kaplan-Meier survival curves were plotted. Differences in OS and PFS between the irAEs and non-irAEs cohorts were compared using the Log-Rank test. We also performed univariate and multivariate COX regression analyses to investigate whether irAEs were independent prognostic factors for OS and PFS in CCA patients. All statistical analyses were performed using the IBM Statistical Package for Social Sciences (SPSS, version 26). A two-sided P<0.05 was considered a significant difference.

## 3 Results

### 3.1 Clinical and demographic characteristics of all included CCA patients

**Figure 1:**
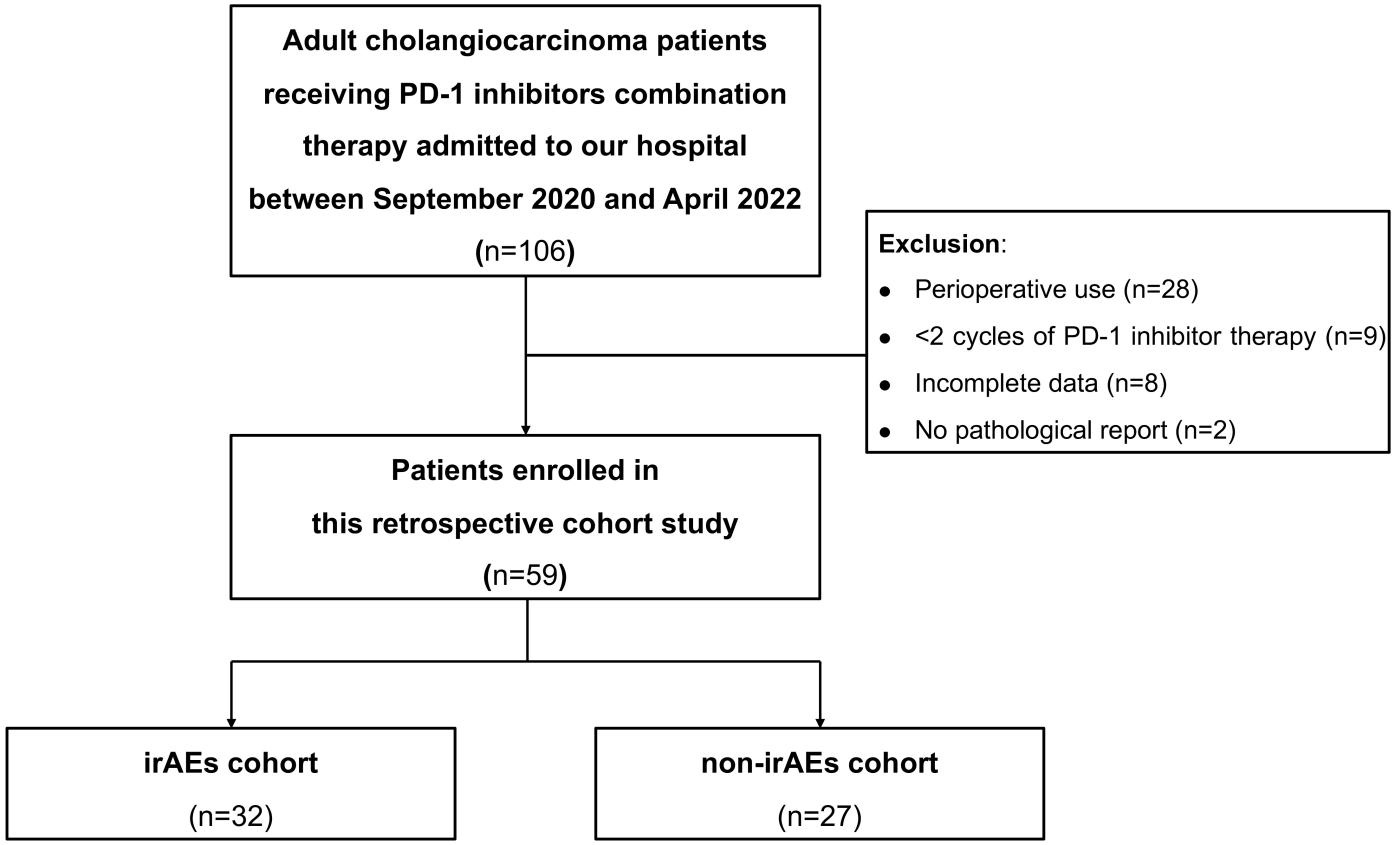
The flow chart of this study. irAEs, immune-related adverse events.

A total of 106 CCA patients receiving PD-1 inhibitors combination therapy at our hospital from September 2020 to April 2022 were screened out. After reviewing the electronic medical records system, 28 patients with perioperative treatments, nine patients with less than two cycles of PD-1 inhibitor treatments, eight patients with incomplete data, and two patients without pathological diagnosis were excluded. Patients were divided into the irAEs cohort and non-irAEs cohort according to whether they had irAEs or not. Finally, 32 patients were included in the irAEs cohort and 27 patients were enrolled in the non-irAEs cohort. The flow chart is shown in **Figure 1**.

The clinical and demographic characteristics of all CCA patients in the irAEs and non-irAEs cohorts are shown in **Table 1**. Of these, 35 (59.3%) patients were treated with sintilimab, 7 (11.9%) with camrelizumab, 5 (8.5%) with pembrolizumab, 4 (6.8%) with toripalimab, and 8 (13.6%) with tislelizumab. In two cohorts, the mean age was 63.3 and 63.0 years, respectively. Fifteen patients (46.9%) in the irAEs cohort and 15 patients (55.6%) in the non-irAEs cohort were male. The mean body mass index (BMI) in two cohorts was 22.1 and 21.8 kg/m^2^, respectively. No pre-existing autoimmune disease (96.9% vs 96.3%), ECOG PS score of 1 (84.4% vs 92.6%), ICC (81.3% vs 63.0%), HBsAg negative (84.4% vs 77.8%), Child-Pugh A (87.5% vs 88.9%), no history of cirrhosis (78.1% vs 81.5%), AFP ≤20 ng/mL (96.9% vs 100.0%), CEA ≤5 ng/mL (62.5% vs 55.6%), CA125 >35 U/mL (56.3% vs 66.7%), CA19-9 >37 U/mL (78.1% vs 74.1%), multiple tumors (75.0% vs 77.8%), lymph node metastasis (81.3% vs 81.5%), AJCC stage IV (62.5% vs 63.0%), PD-1 inhibitors combination therapy as first-line treatment (87.5% vs 96.3%), and PD-1 inhibitors in combination with chemotherapy (62.5% vs 81.5%) were the majority of CCA patients enrolled. There were no statistical differences in most clinical and demographic characteristics between the two cohorts (P>0.05), except for total bilirubin level (P=0.026) and relapse (P=0.016).

**Table 1:** Clinical characteristics of CCA patients with and without irAEs.

Variables	irAEs cohort (N=32)	non-irAEs cohort (N=27)	P-value
Age, mean ± SD, years	63.3 ± 9.1	63.0 ± 8.8	0.903
Sex (male)	15 (46.9%)	15 (55.6%)	0.506
BMI, mean ± SD, kg/m^2^	22.1 ± 2.6	21.8 ± 3.2	0.667
Preexisting autoimmune disease	1 (3.1%)	1 (3.7%)	1.000
ECOG PS			0.570
0	5 (15.6%)	2 (7.4%)	
1	27 (84.4%)	25 (92.6%)	
Primary tumor site			0.115
Intrahepatic	26 (81.3%)	17 (63.0%)	
Extrahepatic	6 (18.8%)	10 (37.0%)	
HBsAg positive	5 (15.6%)	6 (22.2%)	0.517
Platelets, mean ± SD, 10^9^/L	188.3 ± 76.7	220.1 ± 61.9	0.090
Albumin, mean ± SD, g/L	39.7 ± 4.7	40.1 ± 4.5	0.727
Total bilirubin, median (IQR), μmol/L	10.6 (7.3, 16.9)	14.7 (10.0, 21.8)	**0.026**
ALT, median (IQR), U/L	22.0 (16.3, 38.5)	24.0 (13.0, 49.0)	0.982
AST, median (IQR), U/L	29.0 (21.3, 42.5)	26.0 (20.0, 42.0)	0.692
Child-Pugh stage			1.000
A	28 (87.5%)	24 (88.9%)	
B	4 (12.5%)	3 (11.1%)	
Cirrhosis	7 (21.9%)	5 (18.5%)	0.750
AFP, ng/mL			1.000
>20	1 (3.1%)	0 (0.0%)	
≤20	31 (96.9%)	27 (100.0%)	
CEA, ng/mL			0.589
>5	12 (37.5%)	12 (44.4%)	
≤5	20 (62.5%)	15 (55.6%)	
CA125, U/mL			0.414
>35	18 (56.3%)	18 (66.7%)	
≤35	14 (43.8%)	9 (33.3%)	
CA19-9, U/mL			0.716
>37	25 (78.1%)	20 (74.1%)	
≤37	7 (21.9%)	7 (25.9%)	
Tumor number			0.803
Single	8 (25.0%)	6 (22.2%)	
Multiple	24 (75.0%)	21 (77.8%)	
Extrahepatic metastasis	13 (40.6%)	13 (48.1%)	0.562
Lymph node metastasis	26 (81.3%)	22 (81.5%)	0.982
Relapse	14 (43.8%)	4 (14.8%)	**0.016**
AJCC stage			0.971
III	12 (37.5%)	10 (37.0%)	
IV	20 (62.5%)	17 (63.0%)	
MSI status			0.741
High	0 (0.0%)	0 (0.0%)	
Stable	6 (18.8%)	6 (22.2%)	
Unknown	26 (81.2%)	21 (77.8%)	
Treatment line			0.460
First-line	28 (87.5%)	26 (96.3%)	
Second-line and beyond	4 (12.5%)	1 (3.7%)	
Treatment strategy			0.096
Combine with chemotherapy	20 (62.5%)	22 (81.5%)	
Combine with multi-target TKIs	5 (15.6%)	0 (0.0%)	
Combine with chemotherapy and multi-target TKIs	7 (21.9%)	5 (18.5%)	
PD-1 inhibitors			0.208
Sintilimab	19 (59.4%)	16 (59.3%)	
Camrelizumab	4 (12.5%)	3 (11.1%)	
Pembrolizumab	3 (9.4%)	2 (7.4%)	
Toripalimab	0 (0.0%)	4 (14.8%)	
Tislelizumab	6 (18.8%)	2 (7.4%)	

P-values of < 0.05 were shown in bold.

AFP, Alpha-fetoprotein; AJCC, American Joint Committee on Cancer; ALT, alanine aminotransferase; AST, aspartate aminotransferase; BMI, body mass index; CA125, carbohydrate antigen 125; CA19-9, carbohydrate antigen 19-9; CCA, cholangiocarcinoma; CEA, carcinoembryonic antigen; ECOG PS, Eastern Cooperative Oncology Group performance status; HBsAg, hepatitis B surface antigen; IQR, median interquartile range; irAEs, immune-related adverse events; MSI, microsatellite instability; PD-1, programmed cell death protein 1; SD, standard deviation; TKIs, tyrosine kinase inhibitors.

### 3.2 Description of irAEs

In total, 32 (54.2%) patients experienced any- grade irAEs and 4 (6.8%) patients experienced severe (grade 3-4) irAEs. The most common irAEs were thyroid dysfunction (30.5%) and dermatologic adverse events (30.5%), manifesting as hypothyroidism (25.4%), hyperthyroidism (5.1%), rash (22.0%) and pruritus (8.5%). Other irAEs were mainly pneumonia (5.1%), colitis (1.7%), hyperglycaemia (1.7%), pancreatitis (1.7%) and infusion-related reactions (1.7%). The incidence of irAEs is shown in **Table 2**.

**Table 2:** Description of irAEs in CCA patients treated with PD-1 inhibitors in combination with chemotherapy and/or multi-target TKIs.

irAEs category	All patients (N=59)	Patients treated with				
		Sintilimab (N=35)	Camrelizumab (N=7)	Pembrolizumab (N=5)	Toripalimab (N=4)	Tislelizumab (N=8)
Hypothyroidism	15 (25.4)	8 (22.9)	3 (42.9)	2 (40.0)	0 (0.0)	2 (25.0)
Hyperthyroidism	3 (5.1)	3 (8.6)	0 (0.0)	0 (0.0)	0 (0.0)	0 (0.0)
Rash	13 (22.0)	8 (22.9)	2 (28.6)	0 (0.0)	0 (0.0)	3 (37.5)
Pruritus	5 (8.5)	2 (5.7)	1 (14.3)	1 (20.0)	0 (0.0)	1 (12.5)
Pneumonitis	3 (5.1)	2 (5.7)	0 (0.0)	0 (0.0)	0 (0.0)	1 (12.5)
Colitis	1 (1.7)	0 (0.0)	0 (0.0)	1 (20.0)	0 (0.0)	0 (0.0)
Hyperglycemia	1 (1.7)	0 (0.0)	1 (14.3)	0 (0.0)	0 (0.0)	0 (0.0)
Pancreatitis	1 (1.7)	1 (2.9)	0 (0.0)	0 (0.0)	0 (0.0)	0 (0.0)
Infusion-related reactions	1 (1.7)	0 (0.0)	0 (0.0)	0 (0.0)	0 (0.0)	1 (12.5)

Variables are expressed as number of patients (%).

CCA, cholangiocarcinoma; irAEs, immune-related adverse events; PD-1, programmed cell death protein 1; TKIs, tyrosine kinase inhibitors.

### 3.3 Comparison of the efficacy between irAEs and non-irAEs cohorts

**Figure 2:**
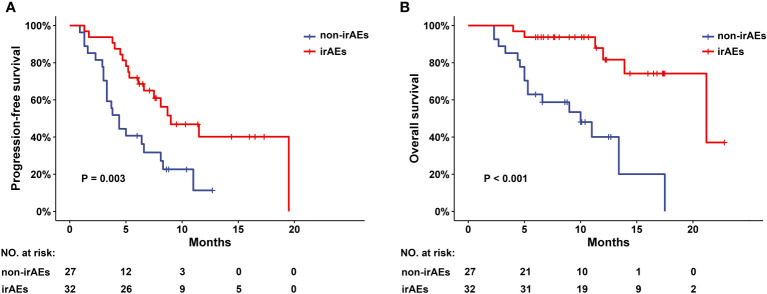
PFS and OS in patients with CCA with or without irAEs.**(A)** Progression-free survival (PFS) and **(B)** overall survival (OS) in CCA patients with or without irAEs after receiving programmed cell death protein 1 (PD-1) inhibitors combination therapy. irAEs, immune-related adverse events; CCA, cholangiocarcinoma.

The median follow-up time was 11.4 (95%CI: 9.1-13.7) months. The ORR was similar in the irAEs and non-irAEs cohorts (21.9% vs 14.8%, P=0.488). The DCR was higher in the irAEs cohort (90.6% vs 70.4%, P=0.047) (**Table 3**). Median PFS was 9.0 (95% CI: 4.9-13.1) months in the irAEs cohort and 4.4 (95% CI: 3.2-5.6) months in the non-irAEs cohorts. Median OS was 21.2 (95% CI: 10.9-31.5) months and 10.0 (95% CI: 5.2-14.8) months in these two cohorts, respectively. Both PFS and OS were better in the irAEs cohort than in the non-irAEs cohort (P=0.003 and P<0.001, respectively) (**Figure 2**).

**Table 3:** Treatment responses of CCA patients with and without irAEs.

Best response	irAEs cohort (N=32)	non-irAEs cohort (N=27)	P-value
CR	0 (0.0%)	0 (0.0%)	0.134^a^
PR	7 (21.9%)	4 (14.8%)	
SD	22 (68.8%)	15 (55.6%)	
PD	3 (9.4%)	8 (29.6%)	
ORR (CR+PR)	7 (21.9%)	4 (14.8%)	0.488^b^
DCR (CR+PR+SD)	29 (90.6%)	19 (70.4%)	**0.047** ^c^

P-values of < 0.05 were shown in bold.

CCA, cholangiocarcinoma; CR, complete response; DCR, disease control rate; irAEs, immune-related adverse events; ORR, overall response rate; PD, progressive disease; PR, partial response; SD, stable disease.

a Best response CR, PR, SD, PD.

b Best response CR + PR vs SD + PD.

c Best response CR + PR + SD vs PD.

### 3.4 Correlation of irAEs with PFS and OS

To investigate whether the differences in patients’ clinical and demographic characteristics affected the results of this study, we performed univariate COX regression analysis, the results showed that total bilirubin >21 μmol/L (HR=2.621, 95% CI: 1.194-5.752, P=0.016), Child-Pugh B (HR=3.316, 95% CI: 1.317-8.353, P=0.011), CEA>5 ng/mL (HR=2.410, 95% CI: 1.256-4.626, P=0.008) and irAEs (HR=0.388, 95% CI: 0.200-0.750, P=0.005) were prognostic factors for PFS. In addition, we also constructed a multivariate COX regression model using the above variables as well as relapse and treatment strategy (P=0.060 in univariate analysis), and the results demonstrated that irAEs were an independent protective factor for PFS (HR=0.435, 95% CI: 0.202-0.934, P=0.033) **Table 4**).

**Table 4:** Univariate and multivariate analyses of prognostic factors for PFS in CCA patients.

Variables	Univariate		Multivariate	
	HR (95% CI)	P-value	HR (95% CI)	P-value
Age (>60 vs ≤60 years)	1.035 (0.526-2.035)	0.921		
Sex (male vs female)	0.647 (0.336-1.244)	0.192		
BMI (>25 vs ≤25 kg/m^2^)	0.499 (0.177-1.412)	0.191		
Preexisting autoimmune disease (yes vs no)	3.075 (0.714-13.241)	0.132		
ECOG PS (1 vs 0)	0.750 (0.312-1.804)	0.521		
Primary tumor site (intrahepatic vs extrahepatic)	0.722 (0.356-1.466)	0.368		
HBsAg (positive vs negative)	0.435 (0.168-1.125)	0.086		
Platelets (>100 vs ≤100 *10^9^/L)	1.881 (0.452-7.833)	0.385		
Albumin (>35 vs ≤35 g/L)	0.516 (0.223-1.194)	0.122		
Total bilirubin (>21 vs ≤21 μmol/L)	2.621 (1.194-5.752)	**0.016**	0.930 (0.306-2.825)	0.899
ALT (>50 vs ≤50 U/L)	1.211 (0.530-2.767)	0.649		
AST (>40 vs ≤40 U/L)	1.181 (0.571-2.443)	0.654		
Child-Pugh stage (B vs A)	3.316 (1.317-8.353)	**0.011**	3.346 (0.925-12.098)	0.066
Cirrhosis (yes vs no)	0.734 (0.321-1.677)	0.463		
AFP (>20 vs ≤20 ng/mL)	3.374 (0.446-25.557)	0.239		
CEA (>5 vs ≤5 ng/mL)	2.410 (1.256-4.626)	**0.008**	1.766 (0.829-3.760)	0.140
CA125 (>35 vs ≤35 U/mL)	1.430 (0.733-2.790)	0.294		
CA19-9 (>37 vs ≤37 U/mL)	0.704 (0.339-1.464)	0.347		
Tumor number (multiple vs single)	0.754 (0.372-1.529)	0.434		
Extrahepatic metastasis (yes vs no)	1.385 (0.725-2.646)	0.324		
Lymph node metastasis (yes vs no)	1.700 (0.661-4.370)	0.271		
Relapse (yes vs no)	0.474 (0.215-1.045)	0.064	1.127 (0.440-2.888)	0.804
AJCC stage (IV vs III)	0.819 (0.422-1.587)	0.553		
Treatment line				
First-line	Reference			
Second-line and beyond	0.390 (0.093-1.635)	0.198		
Treatment strategy				
Combine with chemotherapy	Reference		Reference	
Combine with multi-target TKIs	0.293 (0.070-1.234)	0.094	0.376 (0.083-1.698)	0.203
Combine with chemotherapy and multi-target TKIs	0.368 (0.130-1.044)	0.060	0.481 (0.166-1.399)	0.179
irAEs (yes vs no)	0.388 (0.200-0.750)	**0.005**	0.435 (0.202-0.934)	**0.033**

P-values of < 0.05 were shown in bold.

AFP, Alpha-fetoprotein; AJCC, American Joint Committee on Cancer; ALT, alanine aminotransferase; AST, aspartate aminotransferase; BMI, body mass index; CA125, carbohydrate antigen 125; CA19-9, carbohydrate antigen 19-9; CCA, cholangiocarcinoma; CEA, carcinoembryonic antigen; CI, confidence interval; ECOG PS, Eastern Cooperative Oncology Group performance status; HBsAg, hepatitis B surface antigen; HR, hazard ratio; irAEs, immune-related adverse events; PFS, progression-free survival; TKIs, tyrosine kinase inhibitors.

In addition, univariate COX regression analysis indicated that CEA>5 ng/mL (HR=3.764, 95% CI: 1.481-9.565, P=0.005) and irAEs (HR=0.149, 95% CI: 0.053-0.423, P<0.001) were prognostic factors for OS. We constructed a multivariate COX regression model with the above 2 variables as well as total bilirubin, relapse and treatment strategy. The results showed that irAEs were an independent protective factor for OS (HR=0.133, 95% CI: 0.039-0.452, P=0.001), whereas CEA>5 ng/mL was an independent risk factor for OS (HR=5.718, 95% CI: 1.826-17.902, P=0.003) (**Table 5**).

**Table 5:** Univariate and multivariate analyses of prognostic factors for OS in CCA patients.

Variables	Univariate		Multivariate	
	HR (95% CI)	P-value	HR (95% CI)	P-value
Age (>60 vs ≤60 years)	1.566 (0.606-4.046)	0.354		
Sex (male vs female)	0.969 (0.409-2.294)	0.943		
BMI (>25 vs ≤25 kg/m^2^)	0.750 (0.220-2.554)	0.645		
Preexisting autoimmune disease (yes vs no)	1.077 (0.144-8.089)	0.942		
ECOG PS (1 vs 0)	1.375 (0.387-4.883)	0.622		
Primary tumor site (intrahepatic vs extrahepatic)	0.710 (0.270-1.866)	0.487		
HBsAg (positive vs negative)	0.352 (0.081-1.533)	0.164		
Platelets (>100 vs ≤100 *10^9^/L)	0.786 (0.182-3.391)	0.747		
Albumin (>35 vs ≤35 g/L)	0.578 (0.191-1.749)	0.332		
Total bilirubin (>21 vs ≤21 μmol/L)	1.786 (0.641-4.970)	0.267	0.561 (0.179-1.760)	0.322
ALT (>50 vs ≤50 U/L)	1.774 (0.681-4.621)	0.241		
AST (>40 vs ≤40 U/L)	1.137 (0.440-2.939)	0.791		
Child-Pugh stage (B vs A)	2.379 (0.788-7.182)	0.124		
Cirrhosis (yes vs no)	0.763 (0.253-2.302)	0.632		
AFP (>20 vs ≤20 ng/mL)	5.976 (0.757-47.181)	0.090		
CEA (>5 vs ≤5 ng/mL)	3.764 (1.481-9.565)	**0.005**	5.718 (1.826-17.902)	**0.003**
CA125 (>35 vs ≤35 U/mL)	1.966 (0.790-4.889)	0.146		
CA19-9 (>37 vs ≤37 U/mL)	1.324 (0.445-3.935)	0.614		
Tumor number (multiple vs single)	0.784 (0.311-1.973)	0.605		
Extrahepatic metastasis (yes vs no)	1.452 (0.597-3.532)	0.411		
Lymph node metastasis (yes vs no)	2.751 (0.633-11.950)	0.177		
Relapse (yes vs no)	0.297 (0.087-1.013)	0.053	1.223 (0.292-5.129)	0.783
TNM stage (IV vs III)	0.626 (0.265-1.480)	0.286		
Treatment lines				
First-line	Reference			
Second-line and beyond	0.249 (0.032-1.916)	0.182		
Treatment strategies				
Combine with chemotherapy	Reference		Reference	
Combine with multi-target TKIs	0.235 (0.030-1.813)	0.165	0.534 (0.058-4.948)	0.581
Combine with chemotherapy and multi-target TKIs	0.857 (0.285-2.578)	0.783	1.386 (0.424-4.536)	0.589
irAEs (yes vs no)	0.149 (0.053-0.423)	**<0.001**	0.133 (0.039-0.452)	**0.001**

P-values of < 0.05 were shown in bold.

AFP, Alpha-fetoprotein; AJCC, American Joint Committee on Cancer; ALT, alanine aminotransferase; AST, aspartate aminotransferase; BMI, body mass index; CA125, carbohydrate antigen 125; CA19-9, carbohydrate antigen 19-9; CCA, cholangiocarcinoma; CEA, carcinoembryonic antigen; CI, confidence interval; ECOG PS, Eastern Cooperative Oncology Group performance status; HBsAg, hepatitis B surface antigen; HR, hazard ratio; irAEs, immune-related adverse events; OS, overall survival; TKIs, tyrosine kinase inhibitors.

## 4 Discussion

PD-1 inhibitors are promising anti-tumor drugs that have led to several breakthroughs in the treatment of tumors such as lung cancer and melanoma. A number of clinical trials of PD-1 inhibitors for the treatment of CCA are ongoing, and several case reports and clinical findings suggest that PD-1 inhibitors have therapeutic effects in CCA (11, 12, 29–31). Data from KEYNOTE-158 (11) showed that among 22 patients with advanced CCA with dMMR/MSI-H who received pembrolizumab monotherapy, the ORR was 40.9% (2 CR and 7 PR), and the median PFS and OS were 4.2 (95% CI: 2.1-NR) months and 24.3 (95% CI: 6.5-NR) months, respectively. In another phase II study in 46 patients with advanced BTC (12), 5 patients (11%) responded to nivolumab, all mismatch repair protein-positive tumors. These studies suggest that dMMR/MSI-H may be associated with improved outcomes in CCA patients treated with PD-1 inhibitors, but the evidence is insufficient. Zhang J (31) et al. reported a patient with metastatic ICC who failed first-line chemotherapy and achieved a CR after three cycles of sintilimab monotherapy. However, this patient had a low TMB and was microsatellite stable (MSS). Therefore, there is still a need to explore new biomarkers to predict the effect of PD-1 inhibitors on CCA.

Interestingly, PD-1 inhibitors have the potential to augment normal immune responses while enhancing antitumor immune responses, leading to dysregulation of immune homeostasis and ultimately to irAEs (23). Weber JS et al (32) were the first to report that irAEs of any grade were associated with higher ORR in patients with advanced melanoma receiving nivolumab. Since then, several studies have shown that the occurrence of irAEs is associated with improved ORR, PFS or OS in patients with non-small cell lung cancer (NSCLC) (25, 33–37), gastric cancer (25, 26, 37), renal cell carcinoma (25, 37), urothelial cell carcinoma (38) and hepatocellular carcinoma (28) treated with immunotherapy. However, whether irAEs have a predictive effect in CCA has not been reported. To our knowledge, this is the first evidence that irAEs are associated with better clinical outcomes in patients with advanced CCA receiving PD-1 inhibitors combination therapy. The results of this study showed that among CCA patients receiving PD-1 inhibitors combination therapy, the irAEs cohort had a higher DCR (90.6% vs 70.4%, P=0.047), longer OS (median OS: 21.2 vs 10.0 months, P<0.001) and PFS (median PFS: 9.0 vs 4.4 months, P=0.003) compared to the non-irAEs cohort.

There were statistically differences in total bilirubin levels and relapse between the irAEs and non-irAEs cohorts in the clinical and demographic characteristics of the CCA patients. To assess whether differences in total bilirubin levels and relapse between these two cohorts would affect outcomes, we further performed multivariate COX regression analysis. The results showed that total bilirubin levels did not affect OS and PFS in CCA patients (P=0.322 and 0.899, respectively). Relapse also had no effect on OS and PFS in CCA patients (P=0.783 and 0.804, respectively). Furthermore, irAEs were an independent prognostic factor for PFS in CCA patients (P=0.033), while irAEs and CEA levels were independent prognostic factors for OS (irAEs: P=0.001; CEA levels: P=0.003). The above results suggest that the occurrence of irAEs may predict better outcomes in patients with advanced CCA receiving PD-1 inhibitors combination therapy. Recently, a multicentre retrospective study (39) showed that a higher grade of initial checkpoint inhibitor-related pneumonitis (CIP) was associated with a higher relapse rate of CIP after rechallenge with ICIs in patients with advanced lung cancer. Therefore, we infer that advanced CCA patients with grade 1-2 irAEs may benefit from rechallenge with PD-1 inhibitors, and appropriate studies are needed to verify this inference.

However, the specific mechanisms to explain why irAEs are positively associated with immunotherapy are limited. Studies have shown that a higher BMI associated with improved PFS and OS and a higher incidence of irAEs in cancer patients receiving PD-1/PD-L1 inhibitors (40–42), suggesting that adipose tissue may play a key role in immunotherapy and irAEs. Taken together, the available evidence suggests that this may be related to an increased inflammatory environment due to obesity (42–46). Furthermore, a prospective study of dermatologic adverse events in NSCLC patients receiving PD-1 inhibitors found that tumor and skin tissues shared nine T-cell antigens, and it has been speculated that T cells targeting cancer cells may also target normal tissues with shared antigens (47), which may partially explain the correlation between irAEs and PD-1/PD-L1 inhibitor efficacy. It has also been shown that intestinal flora can modulate innate and acquired immunity in cancer patients in a variety of ways, further influencing the efficacy of PD-1/PD-L1 inhibitors (48–53). While irAEs may also be associated with intestinal flora, studies to date have been limited to CTLA-4 inhibitors. It was found that OS and PFS were improved in patients with Faecal Bacterium and other Firmicutes, but these patients were more likely to develop colitis (54). The specific roles of different microbial species are not yet known and further relevant prospective clinical trials are needed. Finally, high drug clearance (Clearance, CL) has also been suggested as a possible factor to explain the correlation between immunotherapy efficacy and irAEs. One study indicated that high CL was negatively associated with OS and PFS in cancer patients receiving pembrolizumab (P<0.001 and 0.003, respectively) (55). It is possible that high CL promotes the clearance of PD-1 inhibitors, leading to lower blood levels and possibly reduced efficacy. However, this study found no statistical difference in CL between patients with severe irAEs and those with mild irAEs (P = 0.70) (55). Although the CL of PD-1/PD-L1 inhibitors may help explain the correlation between irAEs and the therapeutic effect of PD-1/PD-L1 inhibitors, further evidence is needed.

This study also has some limitations. First, it is a retrospective cohort study, so there may be an inevitable bias for irAEs. Secondly, due to non-randomization, baseline imbalances in patients resulted in the presence of confounding variables. Although we corrected for this using multivariate analysis, validation is needed in prospective, randomized clinical trials. Thirdly, due to the low prevalence of CCA, the number of advanced CCA patients receiving PD-1 inhibitors combination therapy is limited. Therefore, the sample size in this study is small. Finally, the PD-L1 expression and MSI status of most of the patients enrolled were unknown, so we cannot draw any conclusions on the correlation between MSI status, PD-L1 expression and response/survival in advanced CCA patients. In the future, larger prospective studies are needed to validate this finding and identify molecular biomarkers.

## 5 Conclusion

Our study suggests for the first time that irAEs may predict higher DCR and longer OS and PFS in patients with advanced CCA receiving PD-1 inhibitors combination therapy. IrAEs may serve as an effective clinical marker to predict the efficacy of PD-1 inhibitors combination therapy in patients with advanced CCA.

## Data availability statement

The original contributions presented in the study are included in the article/**Supplementary Material**. Further inquiries can be directed to the corresponding authors.

## Ethics statement

The studies involving human participants were reviewed and approved by the First Affiliated Hospital, Zhejiang University School of Medicine (Approval number: IIT20210916A). The patients/participants provided their written informed consent to participate in this study.

## Author contributions

YZ and ZY designed this study. XW and YL collected the data of the participants. YZ, YH contributed to the data analysis. YZ, QZ, and ZY participated in the writing and revision of the manuscript. All authors approved the final version of the article and agreed to be responsible for all aspects of this study.
