# Supplementary material for: Immune-related adverse events correlate with the efficacy of PD-1 inhibitors combination therapy in advanced cholangiocarcinoma patients: A retrospective cohort study
Source: Front Immunol. 2023 Mar 24;14:1141148. doi: 10.3389/fimmu.2023.1141148 (PMC10079946; doi:10.3389/fimmu.2023.1141148)
Supplement: Supplementary file 1 [file Table_1.docx]

**Supplementary TABLE 1** The specific PD-1 inhibitors combination regimens of CCA patients and the clinical presentation and timing of irAEs after treatment.

| **Patient** | **PD-1 inhibitor** | **Combination regimens** | **irAEs** | | |
| --- | --- | --- | --- | --- | --- |
|  |  |  | **Category** | **Grade** | **Timing (month)** |
| #1 | Sintilimab | Lenvatinib | Hypothyroidism | 1-2 | 1.4 |
| #2 | Camrelizumab | Lenvatinib  Gemcitabine  Cisplatin | - | - | - |
| #3 | Pembrolizumab | Lenvatinib  Gemcitabine  Oxaliplatin | - | - | - |
| #4 | Sintilimab | Gemcitabine  Oxaliplatin | Hypothyroidism | 1-2 | 10.6 |
| #5 | Sintilimab | Gemcitabine  Cisplatin | - | - | - |
| #6 | Sintilimab | Gemcitabine  Oxaliplatin | - | - | - |
| #7 | Sintilimab | Gemcitabine  Oxaliplatin | - | - | - |
| #8 | Camrelizumab | Gemcitabine  Oxaliplatin | Rash | 3-4 | 4.0 |
| #9 | Sintilimab | Lenvatinib Gemcitabine  Albumin-bound paclitaxel | - | - | - |
| #10 | Sintilimab | Gemcitabine  Cisplatin | - | - | - |
| #11 | Camrelizumab | Gemcitabine  Oxaliplatin | - | - | - |
| #12 | Toripalimab | Gemcitabine  Cisplatin | - | - | - |
| #13 | Tislelizumab | Lenvatinib | Rash  Hypothyroidism | 1-2 | 3.4  7.6 |
| #14 | Toripalimab | Gemcitabine  Cisplatin | - | - | - |
| #15 | Toripalimab | Gemcitabine  Cisplatin | - | - | - |
| #16 | Camrelizumab | Anlotinib  Capecitabine | - | - | - |
| #17 | Sintilimab | Gemcitabine  Oxaliplatin | Pruritus | 1-2 | 4.0 |
| #18 | Sintilimab | Gemcitabine  Oxaliplatin | - | - | - |
| #19 | Sintilimab | Gemcitabine  Albumin-bound paclitaxel | Hypothyroidism | 1-2 | 1.4 |
| #20 | Pembrolizumab | Lenvatinib  S-1 | Hypothyroidism | 1-2 | 2.6 |
| #21 | Tislelizumab | Gemcitabine  Cisplatin | Hypothyroidism | 1-2 | 2.8 |
| #22 | Sintilimab | Gemcitabine  Albumin-bound paclitaxel | Rash | 1-2 | 2.3 |
| #23 | Camrelizumab | Gemcitabine  Albumin-bound paclitaxel | Hypothyroidism  Hyperglycemia | 1-2 | 4.2  4.2 |
| #24 | Pembrolizumab | Lenvatinib | Pruritus  Hypothyroidism | 1-2 | 3.0  7.0 |
| #25 | Camrelizumab | Lenvatinib | Hypothyroidism  Rash | 1-2 | 2.8  2.8 |
| #26 | Sintilimab | Oxaliplatin  Capecitabine | - | - | - |
| #27 | Sintilimab | Gemcitabine  Cisplatin | - | - | - |
| #28 | Tislelizumab | Gemcitabine  S-1 | Infusion-related reactions | 1-2 | 0.0 |
| #29 | Sintilimab | Gemcitabine  Cisplatin | - | - | - |
| #30 | Sintilimab | Lenvatinib  Gemcitabine  Oxaliplatin | Hyperthyroidism  Rash | 1-2 | 0.5  1.0 |
| #31 | Sintilimab | Gemcitabine  Cisplatin | - | - | - |
| #32 | Tislelizumab | Oxaliplatin  Capecitabine | - | - | - |
| #33 | Pembrolizumab | Gemcitabine  Cisplatin | - | - | - |
| #34 | Sintilimab | Gemcitabine  Cisplatin | - | - | - |
| #35 | Tislelizumab | Gemcitabine  Cisplatin | Rash | 1-2 | 0.0 |
| #36 | Toripalimab | Gemcitabine  Cisplatin | - | - | - |
| #37 | Pembrolizumab | Lenvatinib  Gemcitabine  Cisplatin | Colitis | 3-4 | 6.3 |
| #38 | Sintilimab | Gemcitabine  Cisplatin | Hyperthyroidism | 1-2 | 0.8 |
| #39 | Sintilimab | Gemcitabine  Capecitabine | Rash | 1-2 | 4.0 |
| #40 | Sintilimab | Lenvatinib  Gemcitabine  Cisplatin | Pruritus | 1-2 | 4.0 |
| #41 | Sintilimab | Gemcitabine  Cisplatin | - | - | - |
| #42 | Sintilimab | Oxaliplatin  Capecitabine | Hyperthyroidism  Hypothyroidism | 1-2 | 0.9  3.3 |
| #43 | Tislelizumab | Gemcitabine  Cisplatin | Rash  Pneumonitis | 1-2 | 1.4  1.5 |
| #44 | Camrelizumab | Gemcitabine  Cisplatin | Hypothyroidism  Pruritus | 1-2 | 1.8  2.0 |
| #45 | Sintilimab | Gemcitabine | - | - | - |
| #46 | Sintilimab | Gemcitabine  Oxaliplatin | Rash | 1-2 | 7.0 |
| #47 | Sintilimab | Lenvatinib  Gemcitabine  Oxaliplatin | Pancreatitis | 3-4 | 2.5 |
| #48 | Sintilimab | Oxaliplatin  Capecitabine | Hypothyroidism | 1-2 | 3.7 |
| #49 | Sintilimab | Lenvatinib  Gemcitabine  Oxaliplatin | Rash  Pneumonitis | 1-2 | 0.5  2.1 |
| #50 | Sintilimab | Gemcitabine  Cisplatin | - | - | - |
| #51 | Sintilimab | Lenvatinib  Gemcitabine  Cisplatin | - | - | - |
| #52 | Sintilimab | Gemcitabine  Oxaliplatin | - | - | - |
| #53 | Sintilimab | Gemcitabine  Oxaliplatin | Rash | 1-2 | 1.5 |
| #54 | Sintilimab | Gemcitabine  Oxaliplatin | Rash  Hypothyroidism | 1-2 | 0.5  4.0 |
| #55 | Sintilimab | Gemcitabine  S-1 | Pneumonitis | 1-2 | 2.5 |
| #56 | Tislelizumab | Oxaliplatin  Capecitabine | Pruritus | 3-4 | 0.7 |
| #57 | Tislelizumab | Gemcitabine  S-1 | - | - | - |
| #58 | Sintilimab | Anlotinib | Hypothyroidism | 1-2 | 1.4 |
| #59 | Sintilimab | Lenvatinib  Albumin-bound paclitaxel  S-1 | Rash  Hypothyroidism | 1-2 | 0  6.3 |

**Abbreviations**: CCA, cholangiocarcinoma; irAEs, immune-related adverse events; PD-1, programmed cell death protein 1.
